# Effects of perioperative exercise therapy on cardiorespiratory fitness and postoperative complications in patients with colorectal cancer: a systematic review and meta-analysis

**DOI:** 10.1007/s00520-025-09610-7

**Published:** 2025-06-06

**Authors:** Declan Thomas Hennessy, Kyle J. Miller, Nazib Khan, Sergio Marin Edo, Leo Bell, Pinyadapat Areerob, Stephen Brown, Matthew Wallen

**Affiliations:** 1 Institute of Health and Wellbeing, Federation University Australia, Ballarat, VIC Australia; 2 School of Exercise and Nutrition Sciences, Faculty of Health, Deakin University, Melbourne Burwood Campus, Burwood, VIC 3125 Australia; 3 Grampians Health, Ballarat, VIC Australia; 4 Caring Futures Institute, College of Nursing and Health Sciences, Flinders University, Adelaide, South Australia Australia; 5 Gippsland Integrated Regional Cancer Service (GRICS), Latrobe Regional Health, Traralgon, VIC Australia; 6 School of Psychological Sciences, Monash University, Melbourne, VIC Australia

**Keywords:** Cardiorespiratory fitness, Colorectal cancer, Exercise meta-analysis

## Abstract

**Purpose:**

Improving cardiorespiratory fitness (CRF) before and after colorectal cancer surgery may improve postoperative outcomes. This systematic review and meta-analysis evaluated the effects of perioperative exercise therapy (aerobic, resistance, mind–body, flexibility, or mixed exercise (combined aerobic and resistance)) on CRF, the ventilatory anaerobic threshold (VAT), postoperative complications, and adverse events for patients diagnosed with colorectal cancer undergoing surgery.

**Methods:**

Eligible published randomised controlled trials (RCTs) were identified from an electronic database search (inception—31 May 2020 and updated 16 April 2024). Databases included PubMed, CINAHL, SPORTDiscus, Cochrane Library (CENTRAL), and Web of Science. Pooled standardised mean differences (*SMD*) with 95% confidence intervals (*CI*) were compared, and heterogeneity was assessed using Cochran’s *Q* and *I*^*2*^ statistics.

**Results:**

Twenty-five eligible trials (1385 participants) were included in the meta-analysis. Perioperative exercise demonstrated significant improvements in CRF (*SMD* = 0.28, 95% *CI* = 0.17, 0.38; *p* < 0.05) and the VAT (*SMD* = 0.43, 95% *CI* = 0.23, 0.63;* p* < 0.05) when compared to usual care. No noticeable differences in postoperative complications or adverse events between the groups were found.

**Conclusions:**

Perioperative exercise delivered before and after colorectal cancer surgery significantly improves CRF and the VAT.

**Supplementary Information:**

The online version contains supplementary material available at 10.1007/s00520-025-09610-7.

## Introduction

### Rationale

Colorectal cancer is the third most diagnosed cancer in the world, with more than 1.9 million people diagnosed and 935,000 deaths occurring in 2020 [1]. Additionally, the World Health Organization (WHO) expects a 67% increase in new colon cancer diagnoses and a 58% increase in new rectal cancer diagnoses globally from 2020 to 2040 [2]. Currently, the most frequent curative treatment for colorectal cancer is surgery. Regardless of the staging of cancer, presenting patients have a higher proportion undergoing a form of surgery compared to other treatments (Stage I and II = ~ 97%, Stage III = ~ 98%, Stage IV = ~ 48%) [3]. Although perioperative (before, during, and after treatment) care has significantly improved over the last two decades, postoperative morbidity and mortality rates in colorectal cancer remain an area of concern [4]. Major cancer surgery also comes with the risk of postsurgical declines in functional capacity, irrespective of surgical complications [5, 6]. This is often seen in surgery across a range of cancer populations where declines between 5 and 26% of cardiorespiratory fitness (CRF) are apparent, with some individuals not fully recovering after treatment regimens (e.g. chemotherapy, radiotherapy, and hormone therapy) have concluded [7–11]. Therefore, improving CRF across this continuum is of great importance, as evidence suggests that increases or maintenance in CRF in this cancer cohort are associated with better postoperative outcomes and quality of life (QoL) [12, 13].

Recent studies have contributed to the growing body of evidence supporting the potential efficacy of pre- or postsurgical exercise interventions for improving CRF and functional fitness in colorectal cancer survivors [14–17]. A recent meta-analysis of 48 randomised controlled trials (RCTs) involving 3632 cancer survivors demonstrated the efficacy of exercise training in improving CRF, with a significant increase in V̇O_2peak_ (+ 2.80 mL.min^−1^.kg^−1^) [18]. Another meta-analysis demonstrated that a cohort of 238 colorectal cancer survivors engaging in moderate to high intensity exercise significantly improved physical fitness when compared to those who engaged in lower intensity exercise or received standard care, which included nurse-led phone call follow-ups (*SMD* = 0.59; 95% *CI* 0.25, 0.93; *P* < 0.01) [19]. These results align with a recent meta-analysis by Singh et al., which showed significant improvements in CRF (*SMD* = 0.39), who further advanced the field by showing no significant differences in adverse events with exercise training compared to usual care (*RD* = 0.00; 95% *CI* = − 0.01, 0.01; *p* = 0.92) and that exercise training was well-adhered to (86%). Furthermore, low anaerobic threshold (AT; range 10.1–11.1 mL·min⁻^1^·kg⁻^1^) and V̇O_2peak_ have been shown to predict increased rates of postoperative complications [20].

While the current body of evidence supports exercise training to improve CRF in colorectal cancer survivors, no systematic review has comprehensively synthesised this evidence specifically in this population focusing on CRF, prognostically relevant markers of cardiorespiratory function and reserve (i.e. the VAT), and clinical end-points, including complications [21]. A similar review has been conducted in gastrointestinal cancer more broadly [22], but not with this specific focus on colorectal cancer survivors.

### Objectives

The primary objective of this systematic review and meta-analysis was to compare the change in markers of CRF for colorectal cancer survivors who undertook an exercise training intervention during the perioperative period with those who received usual care or were part of a waitlist control group. Secondary objectives examined the effects of exercise training on safety outcomes, including 30-day postoperative complications and adverse events.

## Methods

This systematic review and meta-analysis were reported in accordance with the Preferred Reporting Items for Systematic Reviews and Meta-analyses (PRISMA) guidelines [23].

### Eligibility criteria

Eligible studies for this systematic review and meta-analysis included RCTs assessing the efficacy of exercise interventions implemented during the perioperative period, defined as the time from diagnosis to planned surgery and from the date of surgery to 6 months following surgery, inclusive of adjuvant therapies. Participants were adults aged 18–80, with no restrictions on sex or source of cancer survivors. Included trials compared exercise interventions to usual care or waitlist controls. CRF was an outcome, assessed using any valid measure of absolute or predicted oxygen uptake, tests estimating CRF, or the 6-min walk test (6MWT). The 6MWT was included explicitly as it is a widely validated measure in clinical settings and is particularly suitable for individuals with low to very low levels of CRF, a common characteristic in clinical trials [24, 25]. Trials were required to report at least two valid CRF measurements (e.g. baseline and follow-up) to assess within-subject changes over time.

Trials were eligible for inclusion if they employed random allocation of participants to intervention and control groups, in accordance with RCT protocols, examination of aerobic (e.g. walking, running, dancing, swimming, or bicycling), resistance (e.g. weight training, training by use of body weight or elastic bands), flexibility, or mind–body exercises in colorectal cancer survivors undergoing major surgery and reporting valid outcome measures for CRF. For the purpose of this review and analysis, mind–body exercise is defined as interventions that include psychological and dietary components that complement the larger exercise component.

Lastly, in studies where not all participants underwent surgery, at least 90% of the overall cohort was required to have undergone surgery for the study to be included.

### Information sources

An initial comprehensive literature search was undertaken from inception until 31 May 2020. An updated search was conducted on 16 April 2024. Databases included PubMed, CINAHL, SPORTDiscus, Cochrane Library (CENTRAL), and Web of Science (see Online Resource 1 for Search Strategy). Reference lists of the included studies and relevant review articles were also searched for potentially eligible studies not captured in the literature search.

### Selection process

Title/abstract and full-text screenings were conducted by two independent assessors (D.H and N.K or L.B). If there were any discrepancies between the two assessors, a third assessor (S.M) decided whether the article was included or excluded. Authors were contacted to request missing data when a complete report was not available for a study. Authors were given 2 weeks to respond and were sent an additional email if there was no response to the first email.

### Data collection process

Two researchers (DH and NK or L.B) extracted the characteristics and outcome data independently, and discrepancies were resolved by discussion and/or consensus with a third researcher (SM). All characteristics and outcome data were recorded using a standardised data collection form.

### Data items

Summary descriptive statistics, including means (M) and standard deviations (SDs) of primary CRF outcomes, were extracted. If SDs were not available but confidence intervals (CI) and interquartile ranges (IQR) were, calculations and approximations were made. The data was then used to calculate the mean changes. Secondary outcomes were also extracted (postoperative outcomes—30-day Clavien-Dindo classification [26] and adverse events).

The primary outcome data of CRF markers (absolute and predicted V̇O_2 max_, V̇O_2peak_ and 6MWT distance) for both the intervention and usual care group were extracted pre- and postintervention and pooled as seen in previous reviews [21, 27]. V̇O_2 max_ is defined as the maximum rate of oxygen uptake evoked during intense exercise, where there is activation of a large muscle mass, whereas V̇O_2peak_ is the highest V̇O2 measurement recorded during a single graded exercise test [28]. In addition, demographic characteristics (sex, gender, chronological age, sample size, tumour classification), intervention characteristics (mode of program, frequency of exercise, length/duration of intervention, type of outcome measure, format of supervision, exercise group adherence, and attrition rate), and methodological characteristics (type of control group, intention-to-treat design) were also extracted. Sample sizes in each condition were also collected to calculate the effect size data of each intervention and control group.

During the data extraction phase, if there was more than one publication linked to the same participant pool, data was extracted from the first published data source.

### Study risk of bias assessment

Two independent reviewers conducted the risk of bias assessment (D.H and N.K) using Version 2 of the Cochrane Collaboration’s Tool for Assessing Risk of Bias in randomised trials (RoB 2 tool) [29]. Discrepancies were resolved by discussion with a third reviewer (S.M). All domains of the RoB 2 tool were classified as low risk, high risk or some concerns.

### Effect measures

Random effects meta-analysis investigated group differences (intervention vs control and different exercise modalities vs control). Standardised mean differences (SMD) (Hedges’ *g*) were calculated for each individual study and pooled into intervention and control groups. Effect sizes in a positive direction determined a more favourable outcome for the intervention, whereas a negative direction determined a more favourable outcome in the respective control group. Postoperative complications and exercise-related adverse events were descriptively reported through counts and percentages.

Confidence intervals (CIs) set at 95% were used for the analysis of any dichotomous outcomes.

Study means ± standard deviations (*SD*) were recorded for key outcome variables and participant characteristics; however, where studies reported standard error of the mean (*SEM*), a manual conversion using the formula *SD* = SEM*√*N* was applied, where *N* is the number of study participants.

### Synthesis methods

Meta-analysis was performed using the Stata/SE software package (version 15.1). Heterogeneity was reported using Cochran’s *Q* and *I*^2^ statistics (the percentage of total variation between studies due to heterogeneity rather than by chance) and was categorised as low (25%), moderate (50%), or high (75%), respectively [30]. Publication bias was presented and examined visually using funnel plots and analysed statistically using Egger’s regression intercept to test for asymmetry. Statistical significance was accepted when *α* < 0.05.

Subgroup analysis was performed by stratifying studies by categorical moderator variables (tumour classification (i.e. colon, rectal, and colorectal), type of control, type of outcome measure, exercise mode, format of supervision, timing of intervention) as separate analyses. Continuous moderator variables (chronological age, length of intervention, duration and frequency of exercise, exercise group adherence, and attrition rate) were investigated by using multiple meta-regression.

### Reporting bias assessment

Risk of bias was assessed using the RoB 2 tool [29]. Criteria included selection of the reported result, measurement of the outcome, missing outcome data, deviations from intended interventions and randomisation process, and overall bias.

### Certainty assessment

The quality of evidence for key findings was assessed using the Grading of Recommendations, Assessment, Development, and Evaluations (GRADE) tool as “very low,” “low,” “moderate,” or “high” [31]. The risk of bias, inconsistency, indirectness, imprecision, and publication bias were assessed for each finding using GRADEpro (https://gradepro.org/). The assessment was performed by one researcher (DH) and confirmed by another (KM). Disagreements were resolved via consensus.

## Results

### Study selection

**Fig. 1:**
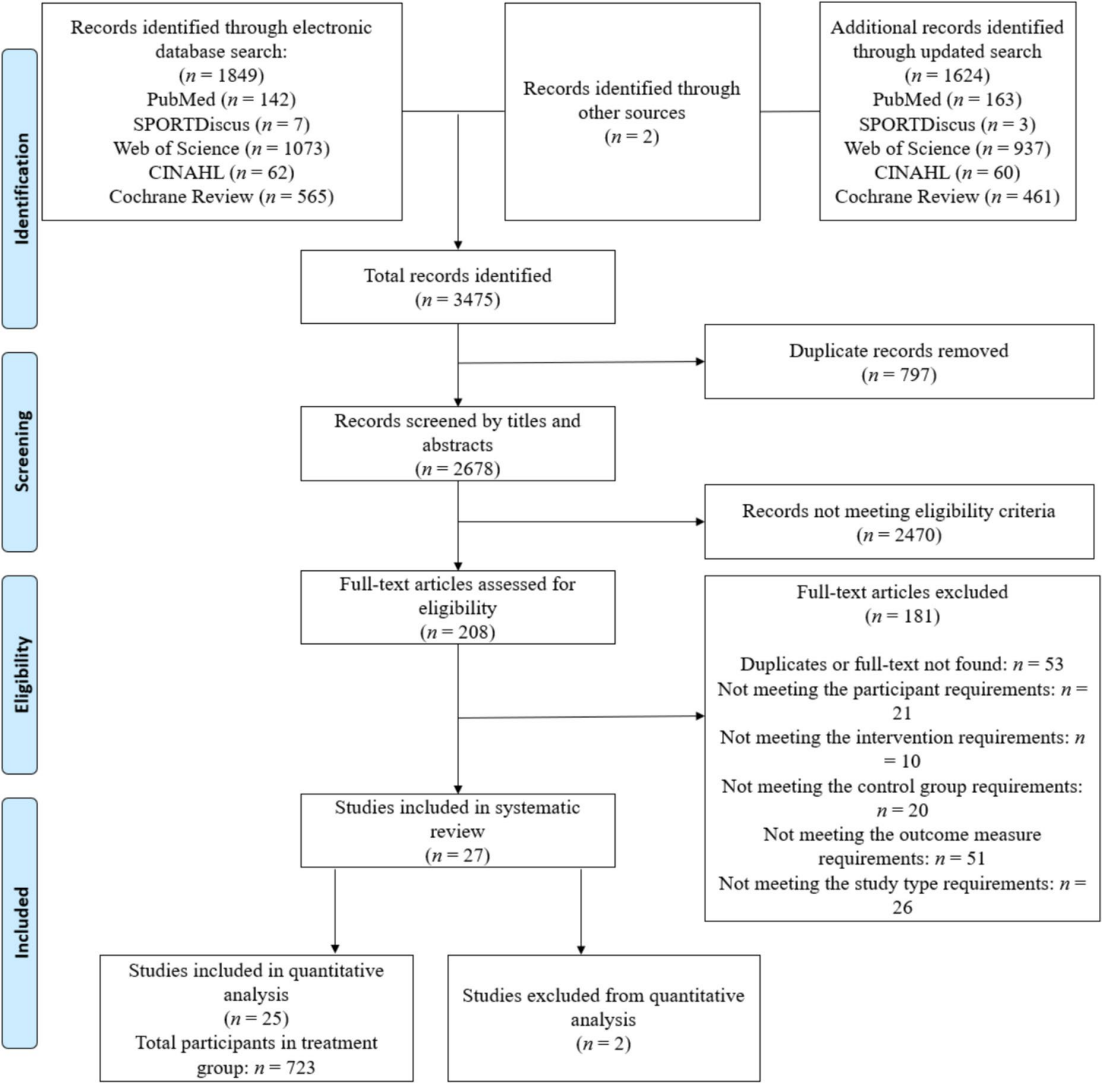
Preferred Reporting Items for Systematic Reviews and Meta-analyses (PRISMA) flow diagram depicting the process of identification and inclusion of selected studies

The initial systematic electronic search resulted in a total of 1849 individual records. An additional 1624 articles were found through the updated search. Another two articles were identified through reference lists from the articles identified in the electronic search. Following the screening of titles and abstracts and subsequent full texts, there were 27 articles that met the eligibility criteria for the systematic review (Fig. 1). Of these 27 studies, 2 RCTs was excluded from the meta-analysis due to the lack of usable outcome data [32, 33]. In addition, no trials were excluded based on the > 90% surgical requirement inclusion criteria outlined within the methods section. Consequently, 25 RCTs were included in the quantitative analysis reporting data on 1385 participants (723 intervention and 662 control) [34–58] (for more details, see Fig. 1). The percentage of agreement between the 1 st and 2nd reviewers for the title and abstract and full-text screening was 90.6% and 87.7%, respectively.

### Study characteristics

#### Demographics

All studies included in the meta-analysis consisted of colon, rectal, or colorectal cancer survivors who completed surgery. At the time of recruitment, cancer survivors in the intervention groups had a mean age of 64.9 (*SD* = 7.2) years. Additionally, mean sample size of the intervention group was 27.8 (*SD* = 26.4). Most trials involved colorectal cancer survivors (*n* = 20, 80%) and the remaining trials included colon (*n* = 3, 12%) and rectal cancer survivors (*n* = 2, 8%). A total of 14 trials (56%) conducted their intervention presurgery (prehabilitation phase), with nine trials (36%) having cancer survivors complete their intervention postsurgery (rehabilitation phase) and a further two trials (8%) occurring during chemotherapy. In addition, 11 of the studies included participants who had undergone neoadjuvant chemoradiotherapy [36, 39, 40, 50, 55, 58–60] or radiation therapy in isolation [41, 47, 61].

#### Measurement of CRF

Across the 25 RCTs, CRF was measured by peak or maximal oxygen uptake (mL.min^−1^.kg^−1^) [38, 42, 46, 49, 51, 53, 55–58, 62], estimated oxygen uptake (mL.min^−1^.kg^−1^) [41, 47], or aerobic exercise tolerance on a treadmill (s) [34]. Exercise capacity was evaluated using 6MWT distance (m) in 11 studies as a surrogate marker of CRF [35–37, 39, 40, 43–45, 50, 52, 54]. A full description of study characteristics can be found in Table 1 and Online Resource 1.

**Table 1:** Study characteristics sorted by cancer type

Author, year	Cancer type	Timing of intervention	Intervention *n*	Intervention mean age (SD)	Sex, female (%)	CRF measurement	Comparator	Comparator *n*	ITT
Bourke (2011)	Colon	Postsurgery	8	67.9 (5.7)	44.4	Aerobic exercise tolerance based on Bruce Protocol (s)	Usual care	9	Yes
Dronkers (2010)	Colon	Presurgery	21	71.1 (6.3)	31.8	Physical Work Capacity 170 Test (O_2_ mL.min^−1^.kg^−1^)	Active control (home-based exercise advice)	20	Yes
Van Vulpen (2015)	Colon	During chemotherapy	10 (M), 7 (F)	58.1 (9.6)	41.2	V̇O_2peak_ (mL.min^−1^.kg^−1^)	Usual care	11 (M), 5 (F)	Yes
Berkel (2022)	Colorectal	Presurgery	28	74 (7)	43	V̇O_2peak_ and V̇O_2_ at VAT (mL.min^−1^.kg^−1^)	Usual care	29	Yes
Bousquet-Dion (2018)	Colorectal	Presurgery	37	74 (median)	19	6MWT (m)	Usual care	39	No
Cantarero-Villanueva (2016)	Colorectal	Postsurgery	21	57.5 (8.0)	38.1	6MWT (m)	Usual care	19	Yes
Carli (2020)	Colorectal	Presurgery	47	78 (median)	47.3	6MWT (m)	Usual care	38	Yes
Chen (2017)	Colorectal	Presurgery	57	67.9 (*SE* = 1.5)	37	6MWT (m)	Usual care	59	No
Christensen (2019)	Colorectal	Postsurgery	19	57.8 (10.4)	63	V̇O_2peak_ (mL.min^−1^.kg^−1^)	Usual care	20	Yes
Courneya (2003)	Colorectal	Postsurgery	62	59.9 (10.7)	45.2	Estimated oxygen uptake based from Modified Balke Treadmill Test—seconds on treadmill	Usual care	30	Yes
Dunne (2016)	Colorectal	Presurgery	19	61 (*IQR* = 56–66)	35	V̇O_2peak_ (mL.min^−1^.kg^−1^)	Usual care	16	No
Falz (2023)	Colorectal	Postsurgery	6	NR for CRC group	NR	V̇O_2Max_ (mL.min^−1^.kg^−1^)	Active control	8	Yes
Fulop (2021)	Colorectal	Presurgery	77	70 (NR)	51.9	6MWT (m)	Usual care	72	Yes
Gillis (2014)	Colorectal	Presurgery	38	65.7 (13.6)	55	6MWT (m)	Usual care	39	No
Karlsson (2019)	Colorectal	Presurgery	10	83.5 (median)	60	6MWT (m)	Usual care	11	No
Kim (2009)	Colorectal	Presurgery	12	55 (15)	36	V̇O_2Max_ (mL.min^−1^.kg^−1^)	Usual care	7	No
Lee (2018)	Colorectal	Postsurgery	38	55.9 (8.8)	52.6	6MWT (m)	Usual care	34	Yes
Mascherini (2020)	Colorectal	Postsurgery	3	72.9 (7.3)	40	6MWT (m)	Usual care	3	No
Molenaar (2023)	Colorectal	Presurgery	123	69.0 (*IQR* = 60–77)	49.6	V̇O_2peak_ (mL.min^−1^.kg^−1^)	Usual care	128	Yes
Morielli (2021)	Colorectal	Presurgery	13	56 (14)	39	V̇O_2peak_ (mL.min^−1^.kg^−1^)	Usual care	12	Yes
Northgraves (2020)	Colorectal	Presurgery	9	64.1 (10.5)	60	6MWT (m)	Usual care	9	Yes
Pesce (2024)	Colorectal	Presurgery	35	68.0 (8.7)	41.7	V̇O_2peak_ (mL.min^−1^.kg^−1^)	Active control	36	No
Pinto, 2011	Colorectal	Postsurgery	19	59.5 (11.2)	60	Estimate of V̇O_2peak_ ("Treadmill test") (mL.min^−1^.kg^−1^)	Active control	24	Yes
Thomsen (2024)	Colorectal	Postsurgery	35	64.0 (NR)	34	V̇O_2Max_ (mL.min^−1^.kg^−1^)	Usual care	17	Yes
Triguero-Cánovas (2023)	Colorectal	Presurgery	23	68.1 (7.7)	30.4	6MWT (m)	Usual care	21	No
Loughney (2016)	Rectal	Presurgery	13	64 (14)	18	V̇O_2peak_ (mL.min^−1^.kg^−1^)	Usual care	12	No
Moug (2019)	Rectal	Presurgery	18	65.2 (11.4)	25	6MWT (m)	Usual care	22	No

*CRC* colorectal cancer, *IQR* interquartile range, *NR* not reported, *SE* standard error

### Results of individual studies

#### Cardio-respiratory fitness

Twenty-five articles (26 groups) were included in the meta-analysis measured changes in CRF. Overall, 21 of the 26 intervention groups showed the exercise intervention to improve CRF in colon/rectal or colorectal cancer patients when compared to a usual care or control group [34, 35, 37–47, 49, 52–58]. Of the 25 RCTs, five studies measured CRF using VAT [38, 42, 49, 55, 57].

Twenty-four of the 26 intervention groups presented data on the frequency of the exercise intervention. The mean frequency of sessions was 4.2 per week (*SD* = 1.6), and the mean length of the intervention was 66.0 days (SD = 48.2). For aerobic exercise, 17 studies (68%) prescribed continuous, low to moderate intensity exercise [34–37, 39–41, 43–45, 47, 51, 52, 54, 56, 58, 62]. Seven studies prescribed interval training, at a moderate-to-high intensity [38, 42, 46, 49, 50, 53, 57]. Four of the six walking interventions presented data on intensity [39, 50, 63, 64]. The walking programs ranged in intensity with two of the studies implementing moderate-intensity programs [39, 45] and two with high-intensity [46, 50]. The duration of the exercise sessions was presented in all studies apart from one [36]. The mean minutes of the session was 62.6 (*SD* = 34.9).

Seven studies (28%) evaluated aerobic as the only type of exercise [38, 39, 46, 49, 51, 53, 55] with another five studies (20%) combining aerobic and resistance training [34, 36, 40, 43, 56]. Seven studies (28%) combined aerobic, resistance, and mind–body training [35, 37, 42, 45, 54, 57, 58]. An additional two studies (8%) combined aerobic, resistance, and inspiratory muscle training (IMT) [48, 50]. The remaining four studies individually examined a combination of aerobic, resistance, and flexibility [44]; a combination of aerobic and IMT [52]; aerobic and flexibility [47]; and aerobic and mind–body [41], respectively.

All studies included in the meta-analysis included aerobic exercise within the intervention, with 15 including a resistance training component to them [34–37, 40, 42–45, 48, 50, 54, 56–58]. Nearly one-quarter of the prescribed aerobic exercise was specifically walking programs (24%) [39, 43–46, 50]. Cycling as the only modality was also frequently prescribed to colorectal cancer survivors (24%) [38, 40, 49, 51, 55, 57].

### Secondary outcomes

#### Postoperative complications

Thirteen studies measured postoperative complications with nine of the studies using the Clavien-Dindo Classification of Surgical Complications [35, 37, 39, 45, 49, 50, 52, 55, 58]. Overall, the exercise intervention groups had a total of 494 participants where postoperative complications were reported (68.3%), while the control/usual care groups had a total of 463 cancer survivors (69.9%). Across the nine studies that measured Clavien-Dindo and specifically outlined the classification, 31, 23, and 14 participants assigned to an exercise intervention experienced Grades 1, 2, or 3 postoperative complications, respectively. There was one Grade 4 complication reported for participants randomised to exercise [58]. For those in the comparator groups, there were 28, 23, 6, and 4 participants who experienced Grades 1, 2, 3, or 4, postoperative complications, respectively. In addition, there was one Grade 5 (death) complication was observed in the pooled control sample.

#### Adverse events

There were 16 studies that quantitatively reported adverse events for the exercise intervention group (64%), and nine studies reported adverse events for the control group (36%). In the exercise intervention group, a total of 28 adverse events were reported across the 16 RCTs, and an additional four adverse events were reported amongst the control groups. There were 11 deaths across all studies (intervention = 7, controls = 4), and no deaths were related to the exercise intervention (Online Resource 1).

### Results of syntheses

**Fig. 2:**
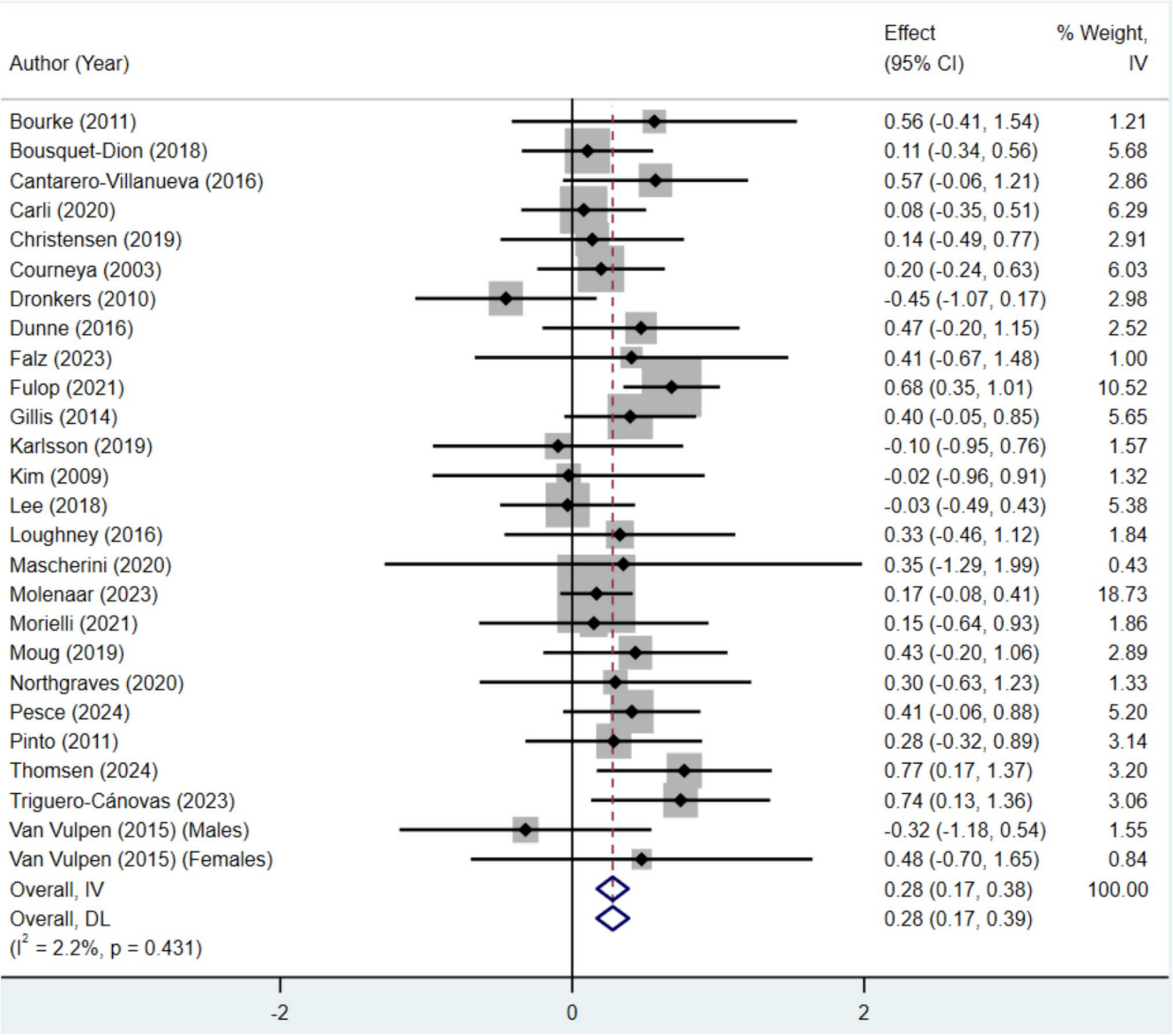
Forest plot of individual and pooled effect size estimates for cardiorespiratory fitness in randomised controlled trials investigating exercise interventions in colorectal cancer survivors. 95% CI, 95% confidence interval, SMD, standardised mean difference

Data from a total of 1385 (723 intervention and 662 control) cancer survivors were extracted and used in the meta-analysis. No publication bias was found through the Egger’s regression test (*p* = 0.922). Compared to usual care, exercise training significantly improved in all CRF outcomes in colorectal, colon, and rectal cancer survivors (*g* = 0.28, 95% *CI* = 0.17, 0.38; *p* < 0.05) (Fig. 2). No significant heterogeneity was identified (*I*^2^ = 2.2%;* p* = 0.431).

Data from a total of 396 (207 intervention and 189 control) cancer survivors were extracted and used in the meta-analysis of VAT [38, 42, 49, 55, 57]. No significant heterogeneity was observed across the intervention groups (*I*^2^ = 0.0%;* p* = 0.715). Compared to the comparator group, exercise interventions significantly improved VAT measures in colorectal, colon, and rectal cancer survivors (Hedges’ *g* = 0.43, 95% *CI* = 0.23, 0.63;* p* < 0.05). Effect sizes and 95% confidence intervals for individual studies are shown in the forest plot (see Online Resource 1 for more details).

**Fig. 3:**
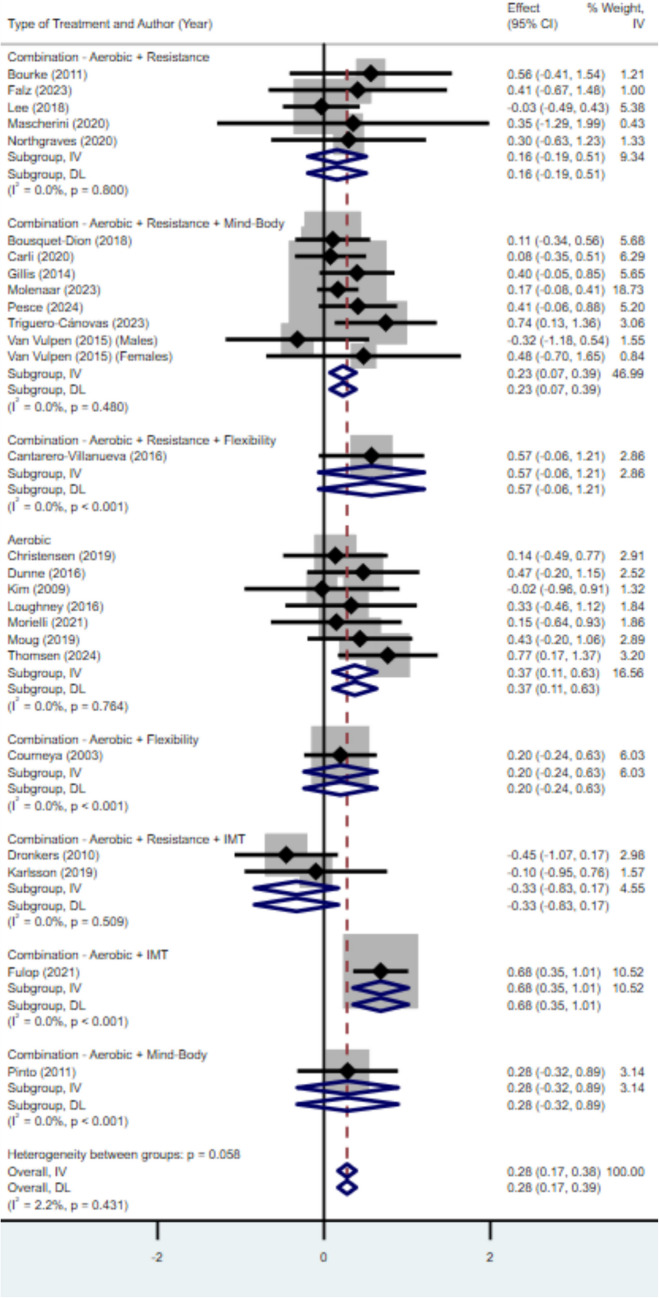
Forest plot of pooled exercise modality effect size estimates for cardiorespiratory fitness in randomised controlled trials investigating exercise interventions in colorectal cancer survivors. 95% CI, 95% confidence interval; SMD, standardised mean difference

Small or small to medium effect sizes were observed for aerobic, combination of aerobic and flexibility, combination of aerobic, resistance and mind–body, combination of aerobic and mind–body, and a combination of aerobic and resistance training. There was a moderate to large effect favouring both the combination of aerobic, resistance, and flexibility exercise (Hedges’ *g* = 0.57) and combination of aerobic and IMT (Hedges’ *g* = 0.68). The results indicate that the combination of aerobic, resistance, and inspiratory muscle training (IMT) may have a small negative effect compared to control conditions (Hedges’ *g* = − 0.33). For more details, see Fig. 3.

### Additional analysis

#### Meta-regression

Meta-regression showed no moderation effect for patient-related factors (intervention and control group chronological age, percentage of females in the intervention and control group). There was also no moderation effect for exercise-specific factors (length of intervention, duration of exercise, exercise group adherence and attrition rate, and total minutes of exercise). Amongst all tested variables, only frequency of exercise emerged as a statistically significant moderator of effect size (*β* = 0.096, *p* = 0.013), indicating that greater exercise frequency was associated with larger treatment effects.

#### Sub-group analysis

Findings from subgroup analysis revealed no significant moderation effects were observed across multiple variables, including intention-to-treat analysis (*p* = 0.471), type of control (*p* = 0.453), format of supervision (*p* = 0.143), and type of outcome measurement (*p* = 0.273). While some individual subgroups demonstrated statistically significant effects (e.g. intention-to-treat: *p* ≤ 0.003; usual care: *p* = 0.000; not supervised: *p* = 0.034), the differences between subgroups for these variables were not statistically significant.

The overall test for heterogeneity between subgroups approached significance (*p* = 0.056) for cancer type, suggesting potential differences in effect sizes across colon and colorectal cancer subgroups. However, no significant heterogeneity was found within the individual subgroups (*p* > 0.05).

In contrast, timing of intervention showed a statistically significant moderation effect (*p* = 0.047), indicating potential differences across subgroups (postsurgery, presurgery, and during chemotherapy). Significant within-subgroup effects were observed for postsurgery (*p* = 0.011) and presurgery (*p* = 0.000), while the during-chemotherapy subgroup was not significant (*p* = 0.873).

Additionally, a significant moderation effect was found for format of exercise (*p* = 0.043), with individual-format interventions demonstrating significant effects (*p* = 0.000), while group-based formats were not (*p* = 0.152). Further information can be found in Online Resource 1.

### Reporting biases and certainty of evidence

All trials in the analysis exhibited at least one risk domain with either a low-risk assessment or some level of concern. For more details, please see Online Resource 1. Specifically, the ‘Deviations from intended interventions’ domain raised ‘some concerns’ in all trials except one. Conversely, the domains of ‘Missing outcome data’ and ‘Measurement of the outcome’ were most frequently rated as low risk, with 81.8% and 86.4% of studies, respectively, receiving this rating. The domains ‘Randomisation process’ and ‘Selection of the reported result’ showed more variation. Notably, 59.1% of trials were categorised as low risk for the ‘Randomisation process’ domain, while 72.7% received a low-risk rating for the ‘Selection of the reported result’ domain. Only one trial was rated as having a high risk of bias overall, while the remaining studies were judged to have some concerns or low risk. Therefore, most trials included in this meta-analysis were deemed to exhibit ‘some concerns’ when assessed for risk of bias.

### GRADE assessment

Measures of CRF included in the *n* = 25 studies indicated ‘high’ certainty and ‘critical’ importance. The outcome of AT was deemed ‘high’ certainty and ‘important’ for importance. For more information, please see Online Resource 1.

## Discussion

The main findings from this systematic review and meta-analysis were that in colorectal cancer survivors (1) perioperative exercise training produced a small but significant improvement in CRF; (2) perioperative exercise significantly improved the AT in survivors; and (3) no significant differences were found in postoperative complications and adverse events between the exercise and comparator groups.

While several reviews have examined the effects of exercise on colorectal cancer survivors [15, 18, 19, 21], this study builds upon and updates prior work in this area, including an earlier review that exclusively included RCTs [13]. By focusing on RCTs, this review ensures a rigorous evaluation of the causal relationship between exercise interventions and outcomes, leveraging the strengths of random allocation, ‘pure’ control groups, and blinding mechanisms [65]. Unlike previous reviews, which often concentrated on specific phases of the treatment cycle such as prehabilitation or rehabilitation [20, 66–69], this analysis provides a more comprehensive examination of perioperative exercise interventions, encompassing prehabilitation, during-treatment, and rehabilitation phases. This broader perspective facilitates an extended understanding of exercise interventions across the treatment continuum and their impact on postsurgical declines, an area of critical importance for surgical patients.

Additionally, this review incorporates novel elements, including an analysis of 30-day postoperative complications, and uniquely includes studies involving participants who underwent radiation or chemoradiation before or after surgery, as well as those with advanced disease, such as liver metastases [55, 70].

### Effectiveness of cardiorespiratory fitness programming in RCTs

The overall pooled analysis showed a small but significant CRF effect when exercise intervention was compared to control groups (*SMD* = 0.28; 95% *CI* = 0.17, 0.38; *p* < 0.05). A total of 21 of the 26 intervention groups showed some improvements in CRF [34, 35, 37–47, 49, 52–58]. Although the observed improvements were not as large as those reported in the meta-analysis by Scott and colleagues (2018), which found a significant increase in V̇O_2peak_ in the exercise therapy group compared to controls (*WMD* = + 2.13; 95% *CI* = 1.58, 2.67; *p* < 0.001) [18], our analysis exclusively included trials where the exercise intervention was implemented within 6 months of surgery. This timing reflects the perioperative focus of our review, where exercise interventions are primarily aimed at mitigating declines in CRF associated with surgical and adjuvant treatment stress. In contrast, larger effects, such as those reported by Scott et al. (2018) and Singh et al. (2022), may be observed in interventions conducted during longer-term recovery periods following treatment, when rebuilding fitness becomes the primary goal [13, 71].

Aerobic exercise prescribed in isolation showed a small to medium but consistent improvement in CRF (*SMD* = 0.37). This could, in part, reflect the principle of specificity, where aerobic exercise training leads to improvements in aerobic capacity as detected by tests such as V̇O_2peak_. While aerobic exercise guidelines are more established, optimal dose–response relationships for other training modalities, such as resistance and inspiratory muscle training (IMT), are still being investigated for cancer survivors [72]. Aerobic, resistance, and IMT-based interventions yielded a pooled effect estimate of *SMD* = − 0.33; however, this should be interpreted cautiously due to marked heterogeneity within the subgroup [48]. Despite the heterogeneity, perhaps the time taken to train the inspiratory muscle group could be used more effectively by substituting another type of exercise or spending more time on aerobic training to elicit CRF improvements.

Exercise training was also found to improve VAT compared to usual care. Several investigations have highlighted the association between preoperative VAT and complications following oncological surgery [73–75] with evidence indicating that a low VAT (10.1–11.1 mL/min^−1^/kg^−1^) may be predictive of postoperative complications [20]. From a clinical perspective, exercise training focused on improving VAT may offer advantages for colorectal cancer survivors, as it allows for individualisation and may require less time compared to conventional CRF training aimed at improving V̇O_2peak_. However, further research is needed to determine whether VAT is a more sensitive marker or associated with a lower rate of non-response compared to V̇O_2peak_. Findings from a recent clinical trial indicate that 4 weeks of HIIT training has the ability to improve the VAT before surgery (+ 1.42 mL/min^−1^/kg^−1^) and has a protective effect 8 weeks postsurgical intervention (+ 2.36 mL/min^−1^/kg^−1^) [76]. In the current study, four out of the five studies (six groups) presented a common trend [38, 42, 49, 57]. All aerobic components of the VAT exercise interventions were completed through interval training protocols and were primarily based on VAT from the baseline CPETs. The bouts prescribed were all moderate to vigorous (%HR and %V̇O_2peak_), and interestingly, five out of the six VAT training groups also had positive effect sizes above SMD = 0.34 in V̇O_2peak_. This therefore indicates that a tailored VAT interval training program may provide optimal CRF improvements in this clinical cohort, where time and efficiency are often critical.

### Effectiveness of cardiorespiratory fitness on 30-day postoperative outcomes in RCTs

Of late, both pre- and postsurgical exercise has been explored for the management of colorectal cancer survivors. This body of research has thus far shown that improving a patient’s CRF can potentially reduce complications following colorectal-based surgery [5, 77]. However, descriptive statistics showed similar trends between the exercise interventions and the control groups when observing 30-day postoperative complications (Clavien-Dindo and undefined complications) [26]. These findings are consistent with a recent review that showed no differences across five studies that reported postoperative complications [78]. Interestingly, when observing the Clavien-Dindo classifications, the control groups had more “life-threatening complications” and deaths (IV = 4, V = 1) compared to exercise groups (IV = 1, V = 0). Although these findings have potential clinical importance, it should be noted that there is a small patient pool (272 and 241, respectively), and therefore, it is difficult to draw any meaningful conclusions.

### Postoperative complications and adverse events reported in RCTs

As seen in previous work, our descriptive analysis revealed no noticeable difference in all postoperative outcomes between the intervention group (34.0%) and the control group (37.8%) [79]. This lack of difference may reflect the focus of our review on perioperative exercise interventions, which target the surgical period. However, emerging evidence advocates for exercise training interventions that improve CRF and functional fitness to be delivered in the preoperative period, with several meta-analyses demonstrating a positive effect on postoperative outcomes after cancer surgery [80–82]. This suggests that while immediate differences in postoperative outcomes may not be evident, preoperative interventions could have a significant impact on long-term recovery and overall surgical success.

Moreover, exercise safety was observed in this review by adverse events. While earlier studies have suggested that participation in exercise interventions may increase the risk of non-serious adverse events by around 19% [83], more recent findings indicate that this risk may be lower in specific cancer populations. For example, colorectal cancer survivors engaging in exercise have been reported to experience a very small adverse event rate of just 1%, suggesting that clinical exercise interventions may be safer in this group than in the general cancer population [21]. Supporting this, a recent meta-analysis involving over 12,000 cancer patients undergoing systemic treatment reported no statistically significant increase in overall adverse events amongst those in the exercise group compared to controls. However, it did identify elevated risks for certain specific events such as thromboses, fractures, and serious adverse events, though the certainty of this evidence was rated very low due to methodological limitations and inconsistent reporting [84]. In line with this, our own findings identified 16 exercise-related adverse events across 522 colorectal cancer survivors (2.3%). This data suggests that while targeted risks may exist, exercise interventions remain broadly safe for colorectal cancer patients, particularly when appropriately prescribed and monitored.

## Limitations

The strengths of this systematic review and meta-analysis lie within the inclusion of only randomised controlled trials and examining effects across the perioperative timeframe (pre- and postsurgical interventions). A limitation of this study is not further exploring what specific exercise type is more effective in improving CRF in colorectal, colon, and rectal cancer survivors perioperatively. However, this particular type of meta-analysis may not be idyllic to determine the most effective exercise intervention. Another limitation noted is that there are limited included studies that measured VAT as a measure of CRF, thus making it difficult to draw any meaningful conclusions from this sub-measure of CRF.

Finally, this meta-analysis did not include any studies that assessed resistance exercise programs in isolation. This may, in part, reflect our focus on CRF as a primary outcome, which tends to prioritise aerobic-based interventions. Additionally, resistance exercise has historically been prescribed with caution for colorectal cancer survivors, particularly in the perioperative stage, due to concerns about the Valsalva manoeuvre being a contraindication. However, over time, numerous exercise trials in cancer survivors have informed clinical recommendations, which now provide safer strategies for prescribing resistance-based exercises [72]. Current guidelines suggest resistance training should be undertaken two or more days a week at 50–70% of 1RM, focusing on all major muscle groups with 8–12 repetitions and 3–4 sets [72, 85]. Future research should explore the benefits and safety of resistance exercise in cancer survivors more comprehensively, particularly in light of these updated guidelines, to enhance the well-being and quality of life of individuals undergoing cancer treatment.

Finally, the included studies within the analysis lacked consistency with the measurement of CRF with both the 6MWT and assessments of oxygen uptake being pooled within the analysis. Although the 6MWT test has been found to be valid in comparison with V̇O_2 max_ in people with low CRF, it appears not to be with adults with average and good age-matched CRF fitness, which may encompass part of the sample size included in this analysis [24].

## Conclusion

Based on the results of this meta-analysis, clinicians and allied health professionals should be encouraged to prescribe exercise to achieve a small yet important improvement in CRF to assist recovery pathways for colorectal cancer survivors. It is recommended that future research should measure AT to better understand cancer survivors’ CRF and to refine exercise prescription in this patient population. Furthermore, future clinical research should begin to assess CRF with more clinically meaningful measurements from cardiopulmonary exercise testing to ensure accurate analysis of CRF in colorectal cancer survivors.

## Supplementary Information

Below is the link to the electronic supplementary material.

Supplementary file1 (DOCX 211 KB)

## Data Availability

All data sourced was available from online journals. If the data was not available, it was sourced from authors if they responded in a timely manner.

## Declarations

### Registration and protocol

This systematic review and meta-analysis were registered in PROSPERO prior to conducting the review (registration number: CRD42020170719).
