# Supplementary material for: Effects of perioperative exercise therapy on cardiorespiratory fitness and postoperative complications in patients with colorectal cancer: a systematic review and meta-analysis
Source: Support Care Cancer. 2025 Jun 6;33(7):551. doi: 10.1007/s00520-025-09610-7 (PMC12144058; doi:10.1007/s00520-025-09610-7)
Supplement: Supplementary file 1 — Supplementary file1 (DOCX 211 KB) [file 520_2025_9610_MOESM1_ESM.docx]

**Table 1:** Intervention characteristics

| Author, Year | Timing of Intervention | Intervention exercise prescription (FITT) | Duration of intervention (days) | Compliance with intervention (%) |
| --- | --- | --- | --- | --- |
| Bourke, 2011 | Post-surgery | 1^st^ 6-weeks:  **F**: Supervised 2/week, Unsupervised 1/week  **I**: AT: Moderate intensity, RT: Between 2 and 4 sets of 8 to 12 repetitions  **T**: 30-min  **T**: Supervised - Combination – Aerobic (Walking, running, rowing, cycling) + Resistance  2^nd^ 6-weeks:  Supervised – 1/week  Unsupervised – 2/week | 84 days | 92 |
| Dronkers, 2010 | Pre-surgery | **F**: 2/week  **I**: AT: Moderate Intensity, RT: 1 x 8-15 reps  **T**: AT: 20-30 mins, RT: NR, IMT: 15 mins  **T**: Supervised Combination - Aerobic (Walking or cycling) + Resistance + IMT | 21 days | 97 |
| Van Vulpen, 2015 | During Chemotherapy | **F**: 5/week  **I**: NR  **T**: AT + RT: 40 min, MB: NR  **T**: Supervised -Combination - Aerobic (Intervals) + Resistance + Mind-Body | 126 days | 89 |
| Berkel, 2022 | Pre-surgery | **F**: 3/week  **I**: Moderate to high intensity  **T**: AT: 40 mins, RT: 20 mins  **T**: Supervised - Combination – Aerobic (Cycle ergometer) + Resistance | 21 days | NR |
| Bousquet-Dion, 2018 | Pre-surgery | **F**: 4.5/week  **I**: Supervised - AT: Moderate intensity  Unsupervised – AT Moderate intensity, RT: 8 exercises – 2 x 8-15 reps  **T**: Supervised - AT: 30 mins, RT: 25 mins, MB: 60 mins  Unsupervised - AT: 30 mins  **T**: Supervised and Unsupervised - Combination - Aerobic (Walking, Jogging, Cycling) + Resistance + Mind-Body | 28 days | 98 |
| Cantarero-Villanueva, 2016 | Post-surgery | **F**: 3/week  **I**: NR  **T**: AT: 10-20 min, RT: 20-30 min, FT: NR  **T**: Supervised - Combination - Aerobic (Walking or jogging) + Resistance + Flexibility | 56 days | 88.36 |
| Carli, 2020 | Pre-surgery | **F**: Supervised – 1/week, Unsupervised - 3/week  **I**: Supervised – Moderate intensity, Unsupervised - NR  **T**: Supervised - AT: 30 min, RT: 25 min  Unsupervised - AT + RT: At least 30 mins  **T**: Supervised and Unsupervised - Combination - Aerobic (Walking) + Resistance + Mind-Body | 28 days | 74 |
| Chen, 2017 | Pre-surgery | **F**: 3/week  **I**: AT - 50% of age-predicted HR, RT - 8-12 repetitions  **T**: AT: 20 min, RT: 20 mins  **T**: Unsupervised - Combination – Aerobic + Resistance + Mind-Body | 28 days | NR |
| Christensen, 2019 | Post-surgery | **F**: Optional how the walking was planned and executed  **I**: Optional how the walking was planned and executed  **T**: 150 mins of walking per week  **T**: Unsupervised - Aerobic - Interval walking | 84 days | 37.5 |
| Courneya, 2003 | Post-surgery | **F**: 4/week  **I**: AT: Moderate intensity  **T**: AT: NR, FT: 20-30 min  **T**: Unsupervised - Combination – Aerobic (any preference of modality) + Flexibility | 112 days | 75.8 |
| Dunne, 2016 | Pre-surgery | **F**: 3/week  **I**: AT: NR  **T**: AT: 30 mins  **T**: Supervised - Aerobic – interval cycling | 28 days | 99 |
| Falz, 2023 | Post-surgery | **F**: 2-3/week  **I**: AT + RT: Moderate to high intensity (HR capped at ~75%max)  **T**: AT + RT: 30 mins (5 min warmup, 20 min circuit, 5 min cooldown)  **T**: Not Supervised – Combination – Aerobic + Resistance (circuit training) | 180 days | 56.4 |
| Fulop, 2021 | Pre-surgery | **F**: Supervised – 1/week, Unsupervised - 6/week  **I**: Supervised – Moderate intensity, Unsupervised - Moderate intensity  **T**: Supervised - AT: 30 mins, IMT: 10-15 min, Unsupervised - AT: 30 mins + IMT: 10-15 min  **T**: Supervised and Unsupervised - Combination - Aerobic (walking or jogging) + IMT | 24-42 days | NR |
| Gillis, 2014 | Pre-surgery | **F**: 3/week  **I**: AT: starting at 40% of HRR, RT: NR, MB, NR  **T**: AT: 20 mins, RT: 20 mins, MB: 60 min  **T**: Unsupervised - Combination – Aerobic (any preference of modality) + Resistance + Mind-Body | 28 days | 78 |
| Karlsson, 2019 | Pre-surgery | **F**: Supervised - 2.5/week, Unsupervised - 2.5/week  **I**: Supervised – NR, Unsupervised - NR  **T**: Supervised – AT + RT + IMT: 60 min, Unsupervised - AT + RT + IMT: 60 min  **T**: Supervised and Unsupervised - Combination - Aerobic (Interval walking) + Resistance + IMT | 17 days | 97 |
| Kim, 2009 | Pre-surgery | **F**: 7/week  **I**: NR  **T**: AT (Continuous): 20-30 mins  **T**: Unsupervised - Aerobic | 28 days | 74 |
| Lee, 2018 | Post-surgery | **F**: AT + RT: Down to patients’ discretion  **I**: AT + RT: Down to patients’ discretion  **T**: AT + RT: Down to patients’ discretion  **T**: Unsupervised - Combination - Aerobic + Resistance | 42 days | 73.5 |
| Mascherini, 2020 | Post-surgery | **F**: Supervised - 2/week, Unsupervised - 3/week  **I**: AT: NR, RT: 8 exercises - 8-12 reps  **T**: AT: 30 mins, RT: NR  **T**: Supervised and Unsupervised - Combination - Aerobic (Walking) + Resistance | 180 days | 100 |
| Molenaar, 2023 | Pre-surgery | **F**: 3/week  **I**: AT: HIIT (4 x 85-90% peak power), RT: 65-70% indirect 1RM  **T**: AT + RT: 60 mins  **T**: Supervised – Combination - Aerobic (cycling) + Resistance + Mind-body | 28 days | 77.2 |
| Morielli, 2021 | Pre-surgery | **F**: Supervised – 3/week, Unsupervised - 1/week  **I**: Supervised - AT: Intervals at 85% of V̇O_2peak_ and active recovery at 40% of V̇O_2peak_ between intervals  Unsupervised - moderate-to-vigorous- intensity continuous exercise  **T**: Supervised - AT: 5 min w/up, 5-8 intervals every 2 mins with 2-mins active recovery  Unsupervised - 150 mins  **T**: Supervised and Unsupervised - Aerobic – (HIIT – Treadmill + general walking) | 69 days | 82 |
| Northgraves, 2020 | Pre-surgery | **F**: 3/week  **I**: AT: Moderate intensity, RT: NR  **T**: AT: 25 min, RT: NR  **T**: Supervised - Combination - Aerobic (Cycling) + Resistance | 22 days | 89.6 |
| Pesce, 2024 | Pre-surgery | **F**: 7/week – AT: 4/week, RT: 3/week  **I**: AT: 50% of HRR then progressed as tolerated, RT: NR  **T**: AT: 60 mins, RT: NR  **T**: Supervised – Combination - Aerobic (walking or cycling) + Resistance + Mind-body | 28 days | NR |
| Pinto, 2011 | Post-surgery | **F**: 3.5/week  **I**: NR  **T**: AT: 10-30 min, MB: NR  **T**: Unsupervised - Aerobic (walking or cycling) + Mind-body | 84 days | 64.7 |
| Thomsen, 2024 | Post-surgery | **F**: 5/week  **I**: Low to high  **T**: 30-50 mins  **T**: Supervised and Unsupervised - Aerobic (cycling) | 56 days | 66 |
| Triguero-Cánovas, 2023 | Pre-surgery | **F**: AT: 7/week, RT: 3/week  **I**: NR  **T**: AT: 30-60 mins, RT: NR  **T**: Supervised and Unsupervised – Combination - Aerobic (walking or cycling) + Resistance (Bodyweight) + Mind-body | NR | NR |
| Loughney, 2016 | Pre-surgery | **F**: 3/week  **I**: Low-moderate intensity  **T**: 30-40 min  **T**: Supervised – Aerobic – walking or cycling | 63 days | 91 |
| Moug, 2019 | Pre-surgery | **F**: 5/week  **I**: Moderate intensity  **T**: 30 mins  **T**: Unsupervised – Aerobic - Walking | 98 days | 75 |
| F = Frequency, I = Intensity, T = Time, T= Type (FITT), SD = Standard Deviation, ITT = Intention-to-treat analysis, AT = Aerobic Training, RT = Resistance Training, MB = Mind-Body Training, IMT = Inspiratory Muscle Training, HRR = Heart Rate Reserve, NR = Not Reported | | | | |

Search String

**PubMed**: (((((colon cancer[Title/Abstract] OR rectal cancer[Title/Abstract] OR colorectal cancer[Title/Abstract]))) AND ((surgery[Title/Abstract] OR perioperative[Title/Abstract] OR presurg*[Title/Abstract] OR prehab*[Title/Abstract] OR rehab*[Title/Abstract] OR preoperative[Title/Abstract] OR postoperative[Title/Abstract]))) AND ((fitness[Title/Abstract] OR VO2 peak[Title/Abstract] OR VO2 max[Title/Abstract] OR ventilatory threshold[Title/Abstract] OR MVC[Title/Abstract] OR exerc*[Title/Abstract] OR strength[Title/Abstract] OR resistance[Title/Abstract] OR aerobic[Title/Abstract] OR flexibility[Title/Abstract] OR training[Title/Abstract] OR yoga[Title/Abstract] OR tai chi[Title/Abstract] OR qigong[Title/Abstract]))) AND ((RCT[Title/Abstract] OR Randomi*[Title/Abstract] OR clinical trial[Title/Abstract] OR controlled trial[Title/Abstract] OR clinical study[Title/Abstract] OR controlled study[Title/Abstract]))

**Cochrane Library (CENTRAL):** ((colon cancer OR rectal cancer OR colorectal cancer)):ti,ab,kw AND ((surgery OR perioperative OR presurg* OR prehab* OR rehab* OR preoperative OR postoperative)):ti,ab,kw AND ((fitness OR VO2 peak OR VO2 max OR ventilatory threshold OR MVC OR exerc* OR strength OR resistance OR aerobic OR flexibility OR training OR yoga OR tai chi OR qigong)):ti,ab,kw AND ((RCT OR Randomi* OR clinical trial OR controlled trial OR clinical study OR controlled study)):ti,ab,kw

**CINAHL:** ( AB (colon cancer) OR TI (colon cancer) OR AB (rectal cancer) OR TI (rectal cancer) OR AB (colorectal cancer) OR TI (colorectal cancer) ) AND ( AB (surgery) OR TI (surgery) OR AB (perioperative) OR TI (perioperative) OR AB (presurg*) OR TI (presurg*) OR AB (prehab*) OR TI (prehab*) OR AB (rehab*) OR TI (rehab*) OR AB (preoperative) OR TI (preoperative) OR AB (postoperative) OR TI (postoperative) ) AND ( AB (fitness) OR TI (fitness) OR AB (VO2 peak) OR TI (VO2 peak) OR AB (VO2 max) OR TI (VO2 max) OR AB (ventilatory threshold) OR TI (ventilatory threshold) OR AB (MVC) OR TI (MVC) OR AB (exerc*) OR TI (exerc*) OR AB (strength) OR TI (strength) OR AB (resistance) OR TI (resistance) OR AB (aerobic) OR TI (aerobic) OR AB (flexibility) OR TI (flexibility) OR AB (training) OR TI (training) OR AB (yoga) OR TI (yoga) OR AB (tai chi) OR TI (tai chi) OR AB (qigong) OR TI (qigong) ) AND ( AB (RCT) OR TI (RCT) OR AB (Randomi*) OR TI (Randomi*) OR AB (clinical trial) OR TI (clinical trial) OR AB (controlled trial) OR TI (controlled trial) OR AB (clinical study) OR TI (clinical study) OR AB (controlled study) OR TI (controlled study) )

**SPORTDiscus**: ( AB (colon cancer) OR TI (colon cancer) OR AB (rectal cancer) OR TI (rectal cancer) OR AB (colorectal cancer) OR TI (colorectal cancer) ) AND ( AB (surgery) OR TI (surgery) OR AB (perioperative) OR TI (perioperative) OR AB (presurg*) OR TI (presurg*) OR AB (prehab*) OR TI (prehab*) OR AB (rehab*) OR TI (rehab*) OR AB (preoperative) OR TI (preoperative) OR AB (postoperative) OR TI (postoperative) ) AND ( AB (fitness) OR TI (fitness) OR AB (VO2 peak) OR TI (VO2 peak) OR AB (VO2 max) OR TI (VO2 max) OR AB (ventilatory threshold) OR TI (ventilatory threshold) OR AB (MVC) OR TI (MVC) OR AB (exerc*) OR TI (exerc*) OR AB (strength) OR TI (strength) OR AB (resistance) OR TI (resistance) OR AB (aerobic) OR TI (aerobic) OR AB (flexibility) OR TI (flexibility) OR AB (training) OR TI (training) OR AB (yoga) OR TI (yoga) OR AB (tai chi) OR TI (tai chi) OR AB (qigong) OR TI (qigong) ) AND ( AB (RCT) OR TI (RCT) OR AB (Randomi*) OR TI (Randomi*) OR AB (clinical trial) OR TI (clinical trial) OR AB (controlled trial) OR TI (controlled trial) OR AB (clinical study) OR TI (clinical study) OR AB (controlled study) OR TI (controlled study) )

**Web of science:** TS=(colon cancer OR rectal cancer OR colorectal cancer) AND TS=(surgery OR perioperative OR presurg* OR prehab* OR rehab* OR preoperative OR postoperative) AND TS=(fitness OR VO2 peak OR VO2 max OR ventilatory threshold OR MVC OR exerc* OR strength OR resistance OR aerobic OR flexibility OR training OR yoga OR tai chi OR qigong) AND TS=(RCT OR Randomi* OR clinical trial OR controlled trial OR clinical study OR controlled study)


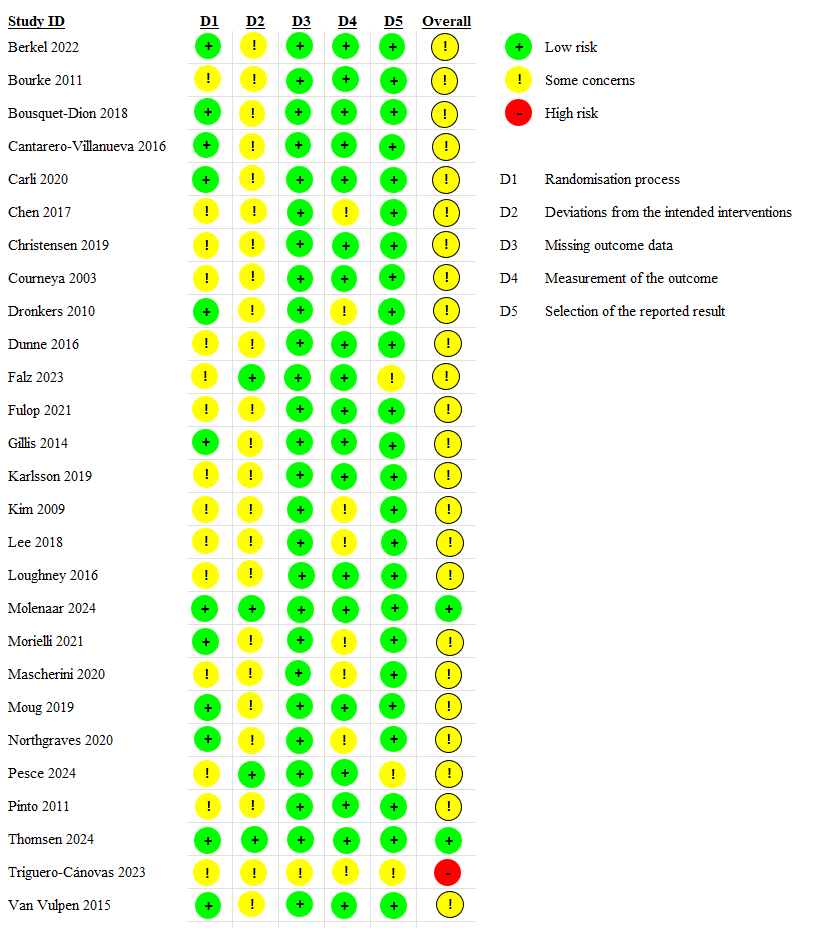


**Figure 1**: Cochrane Collaboration Risk of Bias summary of all included studies in systematic review

**Table 2:** Summary of mortality, post-operative outcomes and adverse events for included articles

|  | | **Aerobic (n=235)** | **Combination – aerobic + resistance + mind-body (n=637)** | **Combination – aerobic + resistance (n=127)** | **Combination – aerobic + resistance + IMT (n=62)** | **Combination – aerobic + resistance + flexibility (n=40)** | **Combination – aerobic + flexibility (n=92)** | **Combination – aerobic + mind-body (n=43)** | **Combination – Aerobic + IMT (n =149)** | **Total** |
| --- | --- | --- | --- | --- | --- | --- | --- | --- | --- | --- |
| **Post-op (Clavien-Dindo)** | Intervention  Control Group | 42/72  38/55 | 33/113  34/103 | 0/0  0/0 | 6/10  2/11 | 0/0  0/0 | 0/0  0/0 | 0/0  0/0 | 17/77  16/72 | 98/272  90/241 |
| **Post-op (undefined or other definition)** | Intervention  Control Group | 6/13  3/8 | 52/178  70/183 | 3/10  4/11 | 9/21  8/20 | 0/0  0/0 | 0/0  0/0 | 0/0  0/0 | 0/0  0/0 | 70/222  85/222 |
| **Adverse clinical events** | Intervention  Control Group | 6/97  3/74 | 3/187  0/54 | 0/47  0/43 | 0/31  0/31 | 3/21  1/19 | 0/62  0/30 | 0/0  0/0 | 0/77  0/72 | 12/522  4/323 |
| **Exercise related adverse events** | Intervention  Control Group | 3/97  0/74 | 4/187  0/54 | 0/47  0/43 | 5/31  0/31 | 4/21  0/19 | 0/62  0/30 | 0/0  0/0 | 0/77  0/72 | 16/522  0/323 |

**
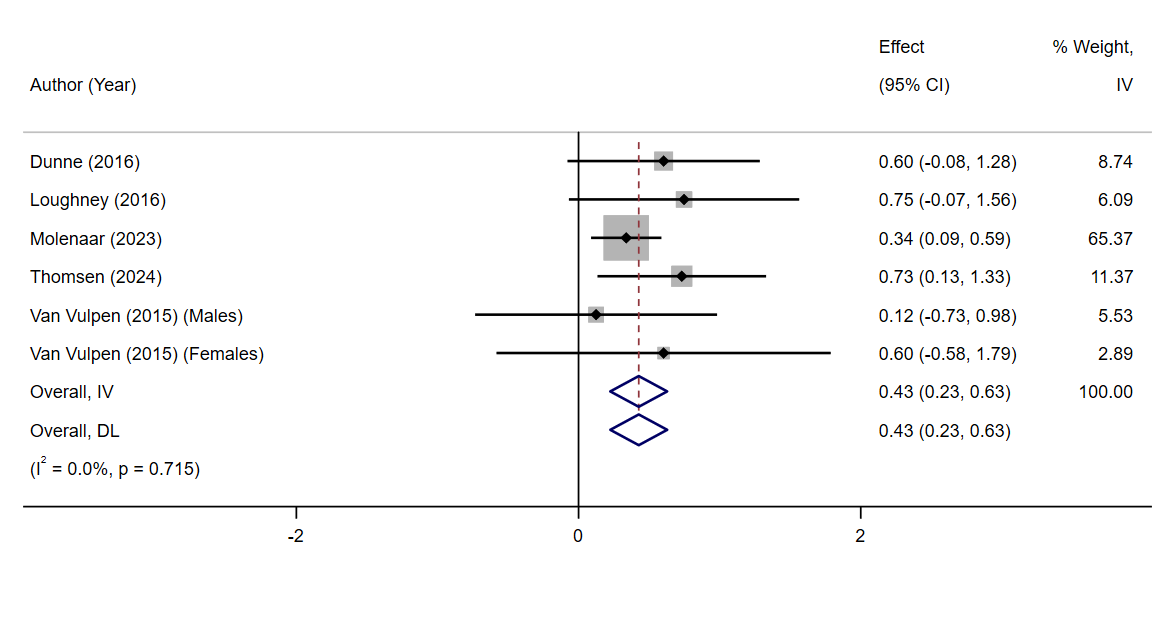
Figure 2**: Forest plot of individual and pooled effect size estimates for VAT in randomised controlled trials investigating exercise interventions in colorectal cancer survivors. 95% CI: 95% confidence interval; SMD: standardized mean difference

**Table 3**: Evidence of GRADE profile

|  | | | | | | | **№ of patients** | | **Effect** | | **Certainty** | **Importance** |
| --- | --- | --- | --- | --- | --- | --- | --- | --- | --- | --- | --- | --- |
| **№ of studies** | **Study design** | **Risk of bias** | **Inconsistency** | **Indirectness** | **Imprecision** | **Other considerations** | **Intervention (aerobic, resistance, mind-body, flexibility, and mixed exercise)** | **Usual Care** | **Relative (95% CI)** | **Absolute (95% CI)** |  |  |
| **Cardio-respiratory fitness (follow-up: median 6 months; assessed with: V̇O2max, V̇O2peak and 6MWT)** | | | | | | | | | | | | |
| 25 | randomised trials | not serious^a^ | not serious | not serious | not serious | none^b^ | 723/723 (100.0%) | 662/662 (100.0%) | **-** | SMD 0.28 SD higher (0.17 higher to 0.38 higher) | ⨁⨁⨁⨁ High | CRITICAL |
| **Cardio-respiratory fitness (follow-up: median 6 months; assessed with: VAT)** | | | | | | | | | | | | |
| 5 | randomised trials | not serious^a^ | not serious | not serious | not serious | none^b^ | 207/723 (28,6%) | 189/662 (25.5%) | **-** | SMD 0.43 SD higher (0.23 higher to 0.63 higher) | ⨁⨁⨁⨁ High | IMPORTANT |

**CI:** confidence interval; **OR:** odds ratio; **SMD**: Standard Mean Difference

^a^ Most information from studies are low or unclear risk of bias. Evidence was therefore not downgraded.

^b^ There was no publication bias. The results were robust. Therefore, the evidence was not downgraded

## Meta-regression and subgroup analysis

| Meta-regression – Patient-related factors | | | | | | |
| --- | --- | --- | --- | --- | --- | --- |
| _ES | Coefficient | Standard Error | t | P>[t] | 95% Conf. Interval | |
| Mean Control Age (yrs) | -.0055532 | .0106293 | -0.52 | 0.606 | -.0275416 | .0164352 |
| % Control Females | .0020708 | .005005 | 0.41 | 0.683 | -.0082827 | .0124244 |
| Mean Exercise Group Age (yrs) | -.0076033 | .0099942 | -0.76 | 0.455 | -.0282779 | .0130714 |
| % Exercise Group Females | .0022745 | .0044649 | 0.51 | 0.615 | -.0069619 | .011511 |

| Meta-regression – Exercise-related factors | | | | | | |
| --- | --- | --- | --- | --- | --- | --- |
| _ES | Coefficient | Standard Error | t | P>[t] | 95% Conf. Interval | |
| Length of the Intervention | .0005131 | .0018471 | 0.28 | 0.784 | -.0033078 | .0043341 |
| Frequency of Exercise | .096168 | .0353959 | 2.72 | 0.013 | -.022762 | .169574 |
| Duration of Exercise Program | -.0004685 | .0021077 | -0.22 | 0.826 | -.0048286 | .0038916 |
| Exercise Group Adherence | -.0030558 | .0047854 | - 0.64 | 0.530 | -.0130075 | .0068959 |
| Exercise Group Attrition Rate | -.0010955 | .0093092 | -0.12 | 0.907 | -.0203086 | .0181177 |
| Total minutes of exercise (duration*frequency*length) | -.0013806 | .000026 | 0.42 | -0.05 | -.0000563 | .0000535 |
| Actual Volume of Exercise  (duration*frequency*length*adherence) | .0000218 | .0000038 | 0.57 | 0.573 | -.0000573 | .0001009 |

Subgroup analysis – Patient-related factors

| Intention to Treat analysis |
| --- |

| Subgroup | z-score (IV) | p-value (IV) | z-score (DL) | p-value (DL) |
| --- | --- | --- | --- | --- |
| Yes | 3.824 | 0.000 | 2.942 | 0.003 |
| No | 3.407 | 0.001 | 3.407 | 0.001 |
| Overall | 5.067 | 0.000 | 4.964 | 0.000 |
| Heterogeneity Between Subgroups | Q = 0.56 | p = 0.455 | F(1,24) = 0.54 | p = 0.471 |

| Cancer Type |
| --- |

| Subgroup | z-score (IV) | p-value (IV) | z-score (DL) | p-value (DL) |
| --- | --- | --- | --- | --- |
| Colon | -0.549 | 0.583 | -0.235 | 0.814 |
| Rectal | 1.560 | 0.120 | 1.878 | 0.837 |
| Colorectal | 5.388 | 0.000 | 5.388 | 0.000 |
| Overall | 5.067 | 0.000 | 4.964 | 0.000 |
| Heterogeneity Between Subgroups | Q = 3.66 | p = 0.056 | F(1,24) = 4.01 | p = 0.057 |

| Type of Control |
| --- |

| Subgroup | z-score (IV) | p-value (IV) | z-score (DL) | p-value (DL) |
| --- | --- | --- | --- | --- |
| Usual Care | 5.008 | 0.000 | 5.008 | 0.000 |
| Active | 1.077 | 0.282 | 0.691 | 0.489 |
| Overall | 5.067 | 0.000 | 4.964 | 0.000 |
| Heterogeneity Between Subgroups | Q = 0.56 | p = 0.453 | F(1,24) = 0.54 | p = 0.469 |

Subgroup analysis – Exercise-related factors

| Timing of intervention |
| --- |

| Subgroup | z-score (IV) | p-value (IV) | z-score (DL) | p-value (DL) |
| --- | --- | --- | --- | --- |
| Post surgery | 1.864 | 0.062 | 1.864 | 0.062 |
| Pre surgery | 1.688 | 0.091 | 1.688 | 0.091 |
| During chemotherapy | 0.159 | 0.873 | 0.159 | 0.873 |
| Overall | 5.067 | 0.000 | 4.964 | 0.000 |
| Heterogeneity Between Subgroups | Q = 10.46 | p = 0.063 | F(5,20) = 2.77 | **p = 0.047** |

| Format of Exercise |
| --- |

| Subgroup | z-score (IV) | p-value (IV) | z-score (DL) | p-value (DL) |
| --- | --- | --- | --- | --- |
| Individual/Group | 0.563 | 0.574 | 0.561 | 0.575 |
| Individual | 5.367 | 0.000 | 5.367 | 0.000 |
| Group | -1.432 | 0.152 | -1.432 | 0.152 |
| Overall | 4.989 | 0.000 | 4.863 | 0.000 |
| Heterogeneity Between Subgroups | Q = 6.28 | p = 0.043 | F(2,22) = 3.77 | p = 0.039 |

| Format of supervision |
| --- |

| Subgroup | z-score (IV) | p-value (IV) | z-score (DL) | p-value (DL) |
| --- | --- | --- | --- | --- |
| Mixed | 4.698 | 0.000 | 3.944 | 0.000 |
| Supervised | 1.753 | 0.080 | 1.485 | 0.138 |
| Not Supervised | 2.124 | 0.034 | 2.124 | 0.034 |
| Overall | 5.067 | 0.000 | 4.964 | 0.000 |
| Heterogeneity Between Subgroups | Q = 3.98 | p = 0.136 | F(2,23) = 2.12 | p = 0.143 |

| Type of outcome measure | | |
| --- | --- | --- |
|  | Z Score | P Value |
| Direct measure | 0.79 | 0.431 |
| Indirect measure | 4.01 | 0.000 |
| Overall | 3.82 | 0.000 |

Overall test for heterogeneity between sub-groups: *p* = 0.152
